# Metropolitan Social Environments and Pre-HAART/HAART Era Changes in Mortality Rates (per 10,000 Adult Residents) among Injection Drug Users Living with AIDS

**DOI:** 10.1371/journal.pone.0057201

**Published:** 2013-02-21

**Authors:** Samuel R. Friedman, Brooke S. West, Enrique R. Pouget, H. Irene Hall, Jennifer Cantrell, Barbara Tempalski, Sudip Chatterjee, Xiaohong Hu, Hannah L. F. Cooper, Sandro Galea, Don C. Des Jarlais

**Affiliations:** 1 Institute of Infectious Disease Research, National Development and Research Institutes, Inc., New York, New York, United States of America; 2 Centers for Disease Control, Atlanta, Georgia, United States of America; 3 Legacy Foundation, Washington, D. C., United States of America; 4 Independent Consultant, Bangalore, India; 5 Department of Behavioral Sciences and Health Education, Emory University, Atlanta, Georgia, United States of America; 6 Department of Epidemiology, Columbia University, New York, New York, United States of America; 7 Baron Edmond de Rothschild Chemical Dependency Institute at Beth Israel Medical Center, New York, New York, United States of America; UCL Institute of Child Health, University College London, United Kingdom

## Abstract

**Background:**

Among the largest US metropolitan areas, trends in mortality rates for injection drug users (IDUs) with AIDS vary substantially. Ecosocial, risk environment and dialectical theories suggest many metropolitan areas characteristics that might drive this variation. We assess metropolitan area characteristics associated with decline in mortality rates among IDUs living with AIDS (per 10,000 adult MSA residents) after highly active antiretroviral therapy (HAART) was developed.

**Methods:**

This is an ecological cohort study of 86 large US metropolitan areas from 1993–2006. The proportional rate of decline in mortality among IDUs diagnosed with AIDS (as a proportion of adult residents) from 1993–1995 to 2004–2006 was the outcome of interest. This rate of decline was modeled as a function of MSA-level variables suggested by ecosocial, risk environment and dialectical theories. In multiple regression analyses, we used 1993–1995 mortality rates to (partially) control for pre-HAART epidemic history and study how other independent variables affected the outcomes.

**Results:**

In multivariable models, pre-HAART to HAART era increases in ‘hard drug’ arrest rates and higher pre-HAART income inequality were associated with lower relative declines in mortality rates. Pre-HAART per capita health expenditure and drug abuse treatment rates, and pre- to HAART-era increases in HIV counseling and testing rates, were weakly associated with greater decline in AIDS mortality.

**Conclusions:**

Mortality among IDUs living with AIDS might be decreased by reducing metropolitan income inequality, increasing public health expenditures, and perhaps increasing drug abuse treatment and HIV testing services. Given prior evidence that drug-related arrest rates are associated with higher HIV prevalence rates among IDUs and do not seem to decrease IDU population prevalence, changes in laws and policing practices to reduce such arrests while still protecting public order should be considered.

## Introduction

Antiretroviral therapy (ART) can delay or prevent HIV-related mortality for people who inject drugs and who have access to and can adhere to treatment regimens [1]. While understanding individual characteristics is important for clinical decision making, public health strategies require a broader understanding of social environmental processes that shape mortality for high risk groups. These can include service provision adequacy and quality, plus other factors that affect levels of access to ART or treatment adherence. They may also include factors that affect mortality independently of, or in interaction with, ART access and use. Krusi et al. recently called for studies of social and structural determinants of ART access and adherence among injection drug users (IDUs) [2]. This paper aims at a related goal: it explores how characteristics of 86 large US metropolitan areas (MSAs) were associated with changes in AIDS mortality since the pre-HAART period among IDUs living with AIDS [3], [4], [5], [6]. These exploratory analyses were guided by ecosocial, risk environment, and dialectical theories about how social environmental processes interact with individual and group creativity and activities to create health outcomes. Ecosocial theories focus on how these social environmental processes are embodied and incorporated biologically over the life course to shape morbidity or mortality [6]; risk environment theories focus more on qualitative insights into the pathways connecting macrosocial and microsocial environments and their relationships with individual agency [3]; and dialectical theory attempts to understand embodiment, pathways and agency in terms of historically-developing processes including collective organizing and thinking on the part of groups of affected people [5].

Our attention is thus not on individual-level predictors of mortality nor multilevel predictors of individual mortality, but rather on predictors of change in area-level mortality *rates* (per 10,000 adult MSA population) among IDUs living with AIDS. The change in such mortality is a good indicator of relative levels of total system success or failure. This is because change in mortality over a period of about 15 years may result from changes in incidence of initiation into injection drug use (or of other increases in drug injection); from changes in HIV incidence among IDUs due to changes in risk behaviors or network structures among IDUs; from changes in the time from HIV incidence to HIV diagnosis; from changes in rates and timing of entry into treatment after diagnosis; from changes in retention once in care; and from changes in mortality rates of those who remain in care [7].

Our prior research suggested that a number of variables related to economic conditions, racial/ethnic inequalities, public finances, social cohesion, and the population prevalence of arrests of drug users in metropolitan areas are related to HIV prevalence among IDUs or to the population prevalence of IDUs [8], [9], [10], [11], [12], [13]. Most other research, however, has studied individual-level predictors of outcomes. Meditz et al. [14] found that at an individual level both race and geographic region affect clinical outcomes of people newly infected with HIV, and Harrison, Song and Zhang [15] showed that race/ethnicity is related to life expectancy among people with HIV. Rubin, Colen and Link [16] showed that socioeconomic status and race were associated with HIV-related mortality in the US both before and after HAART was introduced. IDUs who are in drug abuse treatment, particularly those in methadone or buprenorphine treatment, are more able to gain access to ART and to survive [17]. In a recent review of the literature on ART access and adherence among IDUs, Krusi et al. [2] suggested that structural variables like social exclusion, housing availability and crowding, health care system variables, access to drug abuse treatment, and drug policy variables, like arrests, are likely to affect ART access and adherence—and thus mortality rates.

This paper analyzes changes in metropolitan-area level mortality among IDUs with AIDS as a function of levels of, and/or changes in, economic conditions, government finances, racial/ethnic structures, social cohesion, interventions such as drug abuse treatment, HIV counseling and testing, hard drug arrest rates, and epidemiologic factors like IDU population prevalence and HIV prevalence rates among IDUs.

## Methods

Our unit of analysis is the metropolitan statistical area (MSA). Our study design is a longitudinal study at the MSA level of analysis. As such, it can be considered an “ecological cohort” study of MSAs as social and epidemiologic units. Given the complex pathways likely to occur within MSAs to create the outcome variable (mortality rate of IDUs living with AIDS per 10,000 adult MSA population), this design has important strengths and limitations that are described in the Discussion section of this paper.

The US Census Bureau defines MSAs as contiguous counties containing a central city of 50,000 people or more that form a socioeconomic unity [18]. Studying HIV epidemics among IDUs at the MSA-level [19] is useful since each MSA has its own epidemic history, HIV prevalence rate, and, among HIV-positives, its own distribution of time-since-infection. The broader project in which these analyses are embedded studies the 96 MSAs that had populations of 500,000 or more in 1992. We limited these analyses to the 86 MSAs that had data on key independent variables. Specifically, all analyses were limited to MSAs reporting data on hard drug arrests, which an earlier analysis demonstrated was related to higher HIV prevalence among injectors in 1998 [8], perhaps for reasons related to the rate of change in AIDS mortality among IDUs.

### Analysis

The advent of HAART in 1996 led to rapid declines in AIDS mortality in the United States as a whole [20]; however, we expected variations in AIDS mortality trajectories for IDUs across MSAs due to differences in when HIV entered their IDU populations, in rates and patterns of HIV transmission thereafter [21], [22], and differences in the timing and extent of effective utilization of ART. Data did not exist to allow us to control these differences directly. Instead, we used AIDS mortality rates during 1993–1995 as a control, and analyzed the rate of decline between then and 2004–2006, a period well after HAART became available. Data on IDUs per 10,000 population (aged 15–64), and on changes in this parameter during the study period, were also used to control for the size of the population at risk. This variable refers to people who injected drugs during the prior year [23].

We analyzed rate of decline rather than the absolute magnitude of the decline because AIDS mortality rates (per 10,000 adult population) varied across MSAs by a ratio of more than ten to one (Table 1) and because our focus was on understanding relative changes in local epidemics rather than on individuals *per se*. We used MSA population (aged 15–64) as the denominator for mortality rate calculations rather than estimates of the number of IDUs in the MSA because (1) estimates of current IDUs were available only through 2002 [23]; (2) during the study period, both IDU numbers and metropolitan populations changed considerably in some MSAs; and (3) ex-IDUs who developed AIDS and then died would contribute to mortality data. Importantly, conducting this analysis on a per capita basis means that we analyze the effectiveness of the total system in preventing AIDS mortality among IDUs.

**Table 1:** Average number IDU living with AIDS, number of AIDS deaths, and AIDS mortality rates of IDUs living with AIDS per 10,000 adult population (age 15–64) in 86 large metropolitan statistical areas in the USA 1993–1995 (Pre-HAART) and 2004–2006 (HAART era), and the AIDS mortality rate ratio between late and early years.

	Average No. Living with AIDS 1993–95	Average No. Living with AIDS 2004–06	Average No. of Deaths 1993–95	Average No. of Deaths 2004–06	AIDS Mortality Rate per 10k Adult Pop. 1993–95	AIDS Mortality Rate per 10k Adult Pop. 2004–06	AIDS Mortality Rate Ratio
**Mean**	447.93	790.85	148.67	51.13	0.96	0.32	0.36
**Median**	116.67	282.00	46.33	16.17	0.60	0.18	0.33
**Range**	10.67–13421.33	27.67–20785.00	4.67–4306.67	1.67–1171.00	0.10–7.28	0.04–1.80	0.14–1.01
**Standard Deviation**	1484.00	2338.00	473.82	133.53	1.23	0.38	0.16
**Pseudo-Confidence Interval for the Mean**	129.66–766.20	289.58–1292.00	47.10–250.27	22.50–79.76	0.69–1.22	0.24–0.40	0.33–0.40
**MSA**							
Akron, OH	10.67	35.33	4.67	3.67	0.10	0.08	0.75
Albany–Schenectady–Troy, NY	223.67	394.33	51.67	12.33	0.90	0.20	0.23
Albuquerque, NM	25.67	55.33	18.33	6.33	0.42	0.12	0.29
Allentown–Bethlehem–Easton, PA	107.33	292.00	22.00	5.67	0.55	0.13	0.23
Ann Arbor, MI	19.67	27.67	4.67	2.00	0.13	0.04	0.35
Atlanta, GA	990.67	1864.33	253.67	93.00	1.07	0.28	0.26
Austin–San Marcos, TX	210.33	387.00	81.67	27.00	1.17	0.26	0.22
Bakersfield, CA	95.33	329.00	27.00	7.67	0.71	0.16	0.22
Baltimore, MD	1915.33	4440.00	574.33	294.00	3.49	1.63	0.47
Bergen–Passaic, NJ	705.67	775.67	216.00	62.67	2.47	0.68	0.28
Birmingham, AL	61.67	124.00	38.00	14.33	0.65	0.22	0.34
Boston, MA—NH	1573.33	2647.67	412.00	164.67	1.07	0.39	0.36
Buffalo–Niagara Falls, NY	152.00	300.00	55.33	9.67	0.73	0.13	0.18
Charleston–North Charleston, SC	103.00	152.33	30.00	12.00	0.84	0.29	0.34
Charlotte–Gastonia–Rock Hill, NC—SC	156.33	318.33	60.33	24.00	0.69	0.21	0.30
Cincinnati, OH–KY—IN	50.00	120.00	28.00	7.00	0.27	0.06	0.22
Cleveland–Lorain–Elyria, OH	154.67	285.33	52.00	17.33	0.36	0.12	0.33
Columbus, OH	64.33	126.33	28.67	12.67	0.29	0.11	0.39
Dallas, TX	282.33	754.00	129.33	46.00	0.64	0.17	0.26
Dayton–Springfield, OH	33.00	48.33	11.67	3.33	0.18	0.05	0.29
Denver, CO	120.67	258.67	76.00	31.00	0.60	0.20	0.33
Detroit, MI	535.33	818.67	224.00	63.67	0.78	0.21	0.27
El Paso, TX	27.00	79.67	11.67	7.33	0.28	0.16	0.58
Fort Worth–Arlington, TX	251.33	475.67	71.33	25.67	0.72	0.20	0.28
Fresno, CA	74.33	205.67	31.00	13.67	0.60	0.21	0.35
Gary, IN	32.33	76.33	18.67	7.00	0.46	0.16	0.36
Grand Rapids–Muskegon–Holland, MI	31.00	55.67	13.00	5.33	0.20	0.07	0.35
Greensboro–Winston-Salem–High Point, NC	98.00	180.00	45.33	8.33	0.59	0.09	0.16
Greenville–Spartanburg–Anderson, SC	63.67	142.33	27.67	12.33	0.47	0.18	0.39
Harrisburg–Lebanon–Carlisle, PA	78.00	238.00	22.00	5.33	0.55	0.12	0.23
Hartford, CT	718.00	1264.33	151.33	74.00	2.04	0.93	0.46
Honolulu,HI	27.67	78.33	17.00	8.33	0.29	0.14	0.48
Houston, TX	659.00	1424.00	286.67	140.00	1.15	0.43	0.38
Indianapolis, IN	71.00	187.67	34.33	20.67	0.35	0.18	0.52
Jersey City, NJ	758.67	856.00	242.67	64.67	6.16	1.53	0.25
Kansas City, MO–KS	73.00	165.67	40.67	19.67	0.37	0.16	0.42
Knoxville, TN	12.67	60.33	5.67	6.67	0.13	0.13	1.01
Las Vegas, NV–AZ	154.00	289.00	69.0	34.0	0.93	0.27	0.29
Little Rock–North Little Rock, AR	43.67	86.00	11.33	3.67	0.31	0.09	0.28
Los Angeles–Long Beach, CA	856.33	1741.67	429.67	139.67	0.72	0.21	0.29
Louisville, KY–IN	54.33	161.00	22.67	11.33	0.35	0.16	0.46
Memphis, TN–AR–MS	80.33	211.00	39.67	25.67	0.57	0.32	0.57
Middlesex–Somerset–Hunterdon, NJ	359.00	481.00	128.00	36.67	1.74	0.44	0.25
Milwaukee–Waukesha, WI	86.67	171.67	24.67	13.33	0.26	0.13	0.51
Minneapolis–St. Paul, MN–WI	87.67	174.00	36.33	13.67	0.20	0.06	0.32
Monmouth–Ocean, NJ	366.00	387.00	124.00	35.33	1.93	0.46	0.24
Nashville, TN	105.33	349.33	34.67	35.33	0.47	0.38	0.81
Nassau–Suffolk, NY	694.00	1124.67	236.33	35.33	1.35	0.19	0.14
New Haven–Meriden, CT	1058.33	1771.00	242.33	105.33	2.25	0.92	0.41
New Orleans, LA	260.67	611.33	111.67	68.33	1.29	0.84	0.65
New York, NY	13421.33	20785.00	4306.67	1171.00	7.28	1.80	0.25
Newark, NJ	2303.00	2736.67	722.00	222.00	5.56	1.62	0.29
Norfolk–Virginia Beach–Newport News, VA–NC	170.00	398.00	63.67	28.67	0.62	0.26	0.41
Oakland, CA	358.00	599.00	147.67	44.00	1.00	0.26	0.26
Oklahoma City, OK	39.33	105.00	26.00	11.67	0.39	0.15	0.39
Omaha, NE–IA	18.67	64.00	10.00	5.33	0.23	0.10	0.46
Orange County, CA	207.67	375.00	54.33	16.00	0.31	0.08	0.26
Philadelphia, PA–NJ	1787.33	4211.00	476.00	179.00	1.46	0.52	0.35
Phoenix–Mesa, AZ	155.00	416.67	95.00	50.00	0.56	0.20	0.35
Pittsburgh, PA	106.00	203.33	41.00	9.33	0.27	0.06	0.23
Portland–Vancouver, OR–WA	82.33	229.67	47.67	16.33	0.42	0.11	0.27
Providence–Fall River–Warwick, RI–MA	213.67	374.33	49.33	15.33	0.82	0.23	0.28
Raleigh–Durham–Chapel Hill, NC	135.00	303.33	46.67	20.00	0.67	0.21	0.31
Richmond–Petersburg, VA	154.33	289.67	54.67	15.33	0.87	0.21	0.24
Riverside–San Bernardino, CA	317.33	552.00	115.00	33.33	0.64	0.13	0.21
Rochester, NY	352.33	669.33	74.00	19.33	1.04	0.26	0.25
Sacramento, CA	129.00	213.00	63.33	11.33	0.66	0.09	0.14
St. Louis, MO–IL	112.67	239.67	34.67	16.33	0.21	0.09	0.43
Salt Lake City–Ogden, UT	77.67	164.67	23.33	15.00	0.30	0.16	0.52
San Antonio, TX	111.67	304.67	46.00	18.33	0.49	0.16	0.32
San Diego, CA	216.00	535.00	115.33	49.00	0.67	0.25	0.37
San Francisco, CA	775.33	1124.67	399.67	144.67	3.46	1.19	0.35
San Jose, CA	97.00	156.00	41.00	8.00	0.38	0.07	0.18
Scranton–Wilkes-Barre–Hazleton, PA	52.00	111.00	11.67	1.67	0.29	0.04	0.14
Seattle–Bellevue–Everett, WA	123.33	278.67	85.33	30.67	0.56	0.17	0.30
Springfield, MA	242.00	417.00	78.67	34.33	2.01	0.82	0.41
Stockton–Lodi, CA	45.67	132.00	16.33	13.33	0.51	0.31	0.60
Syracuse, NY	127.33	226.33	35.33	6.67	0.73	0.14	0.19
Tacoma, WA	46.33	87.00	18.67	11.67	0.44	0.23	0.51
Toledo, OH	15.67	41.67	7.00	5.00	0.17	0.12	0.70
Tucson, AZ	53.67	119.00	30.00	15.67	0.62	0.25	0.41
Tulsa, OK	27.67	71.00	13.00	8.33	0.27	0.15	0.57
Ventura, CA	15.33	46.00	10.67	3.67	0.23	0.07	0.30
Washington, DC–MD–VA–WV	1391.33	3225.33	421.33	200	1.34	0.53	0.40
Wilmington–Newark, DE–MD	253.00	532.67	72.67	47.67	1.98	1.13	0.57
Youngstown–Warren, OH	15.00	46.33	4.67	2.67	0.12	0.07	0.59

### Dependent Variable and Analysis Equation

We compared CDC's National HIV Surveillance System data on AIDS mortality among IDU with AIDS per 10,000 adult population averaged across 1993–1995 in a given MSA with that averaged across 2004–2006. The conceptual dependent variable was the ratio of mortality in the later ART era period divided by mortality in the earlier pre-HAART period [24]. To avoid well-known statistical problems of correlated error terms, we modeled this using linear regression techniques based on Equation 1:
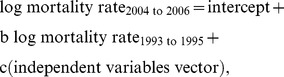
(1)where mortality rate indicates the number of deaths among IDUs with AIDS divided by the total population aged 15–64 for a given MSA, averaged over the indicated years. Since we use standardized regression coefficients as a measure of effect size, Equation 1 would underestimate effects because it includes in the variance of the dependent variable all the variance due to differences in mortality in the earlier period, whereas our interest is in the proportional change in mortality rates. We thus used Equation 2, which differs from Equation 1 only in that we subtracted log mortality rate _1993 to 1995_ from both sides of the equation:
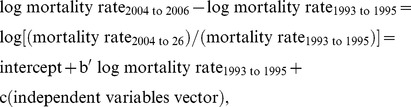
(2)where b′ = b−1.

CDC data on “IDUs living with AIDS” includes people who currently inject drugs *and* people who have stopped injecting. Deaths include people who died for reasons unconnected with HIV or AIDS. By decreasing AIDS deaths, ART probably increased the proportion of people with AIDS who died for unrelated reasons [25].

### Independent Variables

Categories of independent variables, descriptive statistics, and information on data sources are listed in Table 2. For variables where data were not available for the periods 1993–1995 and/or 2000–2002, we used the most recent earlier data for the variable. All of these independent variables are measured for time periods earlier than 2004–2006 in order to reduce issues of reverse causation.

**Table 2:** Descriptive statistics on mortality rates of IDUs living with AIDS per 10,000 adult population (age 15–64) and independent variables during pre-HAART and HAART-era periods.

		Pre-HAART					HAART era				Data Source
	N	Median (Range)	Mean (SD)	1^st^ Quartile	3^rd^ Quartile	N	Median (Range)	Mean (SD)	1^st^ Quartile	3^rd^ Quartile	
AIDS Mortality (Log) (1993–95, 2004–06)	86	−0.52 (−2.26–1.99)	−0.47 (0.89)	−1.16	0.07	86	−1.70 (−3.18–0.59)	−1.57 (0.87)	−2.11	−1.22	CDC*
MSA Adult Population Size (in thousands)*	86	742 (317–6,007)	1,111 (1,035)	483	1,299	86	871 (377–6,429)	1,225 (1,127)	507	1,450	US Census Bureau [64]
IDUs per 10,000 adult population (1993–95, 2000–02)**	86	100.90 (35.84–301.15)	122.17 (63.99)	73.43	164.96	86	101.72 (36.11–320.85)	115.31 (60.60)	66.16	141.49	Brady et al. 2008 [23]
**Economic Conditions**											
Household Gini Coefficient*** (1990, 2000)	86	0.43 (0.38–0.51)	0.43 (0.02)	0.41	0.44	86	0.44 (0.41–0.54)	0.44 (0.02)	0.43	0.45	Harper [65]
Percent Living Below Poverty Level (1990, 2000)	86	11.17 (4.23–26.80)	11.52 (3.82)	9.42	13.17	86	10.45 (5.45–23.81)	11.18 (3.49)	8.74	12.18	US Census Bureau [66], [67]
Unemployment Rate (1993–95, 2000–02)	86	5.45 (2.87–14.93)	5.87 (2.25)	4.30	6.47	84	4.53 (3.07–10.70)	4.69 (1.41)	3.97	4.95	BLS [68]
**Fiscal Conditions**											
Long Term Debt per capita (in constant 2010 dollars) (1992, 2002)	86	3202.86 (932.91–13694.33)	3874.06 (2255.29)	2354.44	4877.33	86	4037.96 (1261.96–9584.15)	4456.27 (1863.47)	3000.60	5584.50	US Census Bureau [69]
Health Care Expenditure per capita in 2010 $'s (1992, 2002)	86	83.82 (3.18–331.06)	93.25 (61.11)	45.55	144.47	86	115.29 (3.62–501.33)	128.62 (93.67)	55.98	194.13	US Census Bureau [69]
**Housing Conditions**											
Crowding (>1 occupant per room) (1990, 2000)	86	0.03 (0.01–0.21)	0.05 (0.04)	0.02	0.06	86	0.04 (0.01–0.23)	0.06 (0.05)	0.03	0.07	US Census Bureau [70], [71]
**Racial/Ethnic**											
Black/White Poverty Disparity (1990, 2000)	86	3.50 (0.70–7.18)	3.48 (1.03)	2.88	4.18	86	3.46 (1.10–6.94)	3.49 (0.87)	2.95	4.18	US Census Bureau [72]
Black/White Unemployment Disparity (1990, 2000)	86	2.63 (0.88–4.95)	2.60 (0.66)	2.09	2.94	86	2.67 (1.10–4.89)	2.70 (0.58)	2.38	2.98	US Census Bureau [72]
Black/White Dissimilarity Index (1990, 2000)	81	63.69 (37.52–89.95)	63.81 (12.06)	55.43	73.09	81	60.91 (31.81–84.72)	60.01 (12.37)	52.12	69.11	Mumford Center Data [73]
Hispanic/White Dissimilarity Index (1990, 2000)	81	39.28 (21.49–66.76)	40.51 (11.40)	30.58	49.97	81	46.32 (26.76–66.68)	45.46 (9.44)	38.02	51.94	Mumford Center Data [73]
**Social Cohesion**											
Religious Membership per 10,000 adult population (1990, 2000)	86	1280.60 (294.65–4945.15)	1676.90 (1067.02)	850.48	2155.21	86	1108.34 (232.49–4482.47)	1450.45 (975.52)	717.36	1892.43	ARDA [74]
Congregations per 10,000 adult population (1990, 2000)	86	6.30 (3.39–15.91)	7.17 (2.92)	5.09	8.31	86	5.60 (3.13–15.50)	6.49 (2.59)	4.79	7.51	ARDA [74]
**Interventions**											
Hard Drug Arrests per 10,000 adult population (1993–95, 2000–02)	86	10.76 (0.80–60.16)	14.52 (12.20)	5.65	19.51	86	9.93 (0.59–56.84)	12.85 (9.86)	6.63	16.35	FBI [75]
HIV Counseling and Testing Coverage for IDU (1993–95, 2000–02)	79	6.43 (0.81–36.59)	8.13 (7.22)	3.59	9.32	81	5.38 (0.23–45.10)	8.23 (8.30)	3.03	10.54	Tempalski et al. 2010 [47]
Drug Treatment Coverage for IDU (1993–95, 2000–02)	81	6.05 (1.00–15.60)	6.99 (3.81)	4.15	10.20	81	7.70 (0.80–22.35)	8.05 (4.28)	4.90	9.80	Tempalski et al. 2010 [47]
Methadone Maintenance Coverage for IDU (1993 & 1995, 2000 & 2002)	77	3.47 (0.00–11.46)	3.80 (2.34)	2.03	5.18	74	6.23 (0.17–17.26)	6.58 (3.46)	3.98	8.76	Brady et al. [23], TEDS [76], UFDS/N-SSATS [77]
**Epidemiologic Factors**											
HIV Prevalence IDU (1993–95, 2000–02)	86	6.27 (2.38–37.98)	9.38 (7.47)	4.06	11.39	86	3.94 (1.99–22.08)	5.76 (4.07)	3.11	7.13	Tempalski et al. 2009 [78]
Drug-related Overdose Death rate per 10,000 adult population (1993–95, 2000–02)	86	0.26 (0.01–1.47)	0.34 (0.29)	0.12	0.48	86	0.26 (0.02–0.99)	0.26 (0.21)	0.15	0.46	Cooper et al. 2008 [79]

**Note:** ARDA – Association of Religious Data Archives; BLS – Bureau of Labor Statistics; CDC – Centers for Disease Control and Prevention; FBI – Federal Bureau of Investigation; SAMSHA N-SSATS – Substance Abuse and Mental Health Services National Survey of Substance Abuse Treatment Services. We used intercensal estimates of population aged 15–64 [66], [67].

* US AIDS Mortality Surveillance Data for 1991–2006 received by special data request (2009) from the US Department of Health and Human Services, Centers for Disease Control and Prevention, National Center for HIV and TB Prevention.

** Estimates of IDUs per 10,000 adult population are estimates of the proportion of the adult population who injected drugs in the prior year.

*** Gini coefficients are measures of the extent to which distributions of resources within a population would need to change to create equality. Zero represents equality, 1 represents maximum inequality. The household Gini used here presents data on inequality in household incomes.

Independent variables fall into seven domains that are consistent with ecosocial, dialectical and risk environment theories [3], [4], [5], [6]. *Economic conditions* include measures of poverty, unemployment and income inequality (Gini coefficient), which is in accordance with a broad social determinants of health perspective [26], [27], [28], [29]. *Fiscal conditions* include long-term debt (per capita) of governments in the MSA, and per capita public health care expenditures. Both of these may influence the extent of access to ART. *Housing conditions* were suggested by Krusi et al. [2] as a potential predictor of ART access and adherence for drug users. *Racial/ethnic variables* measure exclusion and inequality on dimensions of structural racism. Some available measures of White/Hispanic disparities are not included in the analysis because of geographic differences among Hispanic subgroups. *Social cohesion* measures metropolitan area characteristics like religiosity that may be proxies for social factors contributing social support for IDUs in getting access to medications and adhering to medical regimens. *Interventions* for IDUs take several forms. “Hard drug” arrests (those for possession of cocaine, opioids or amphetamine) are associated with higher HIV prevalence [8] and may make it more difficult for IDUs to remain adherent [2]. Higher HIV counseling and testing rates make it more likely that IDUs with HIV get diagnosed early in disease progression, which should decrease their mortality rates. Drug treatment coverage, and particularly methadone coverage, are associated with improved medical care in general and with greater access to ART and adherence to dosing schedules [30], [31]. *Epidemiologic factors* include HIV prevalence among IDUs and overdose death rate per 10,000, which should directly affect mortality rates among IDUs living with AIDS.

### Statistical Analysis

Analyses were designed to be exploratory and descriptive rather than to test hypotheses. We had good theoretical reasons for including a large number of independent variables. Furthermore, neither prior research nor theory could guide us on whether independent variables should be analyzed in terms of their baseline values, their rate of change during the period of interest, or their absolute magnitude of change over the period of interest, our exploratory analyses included entering variables on a given topic in these different functional forms. For this same reason, we explored whether including the baseline value of an independent variable, the baseline value plus a change measure, or a change measure alone would produce a more parsimonious model.

Since we studied the 86 US MSAs with populations of 500,000 or more in 1992 that had data available on drug arrest rates, our sample is a completely-enumerated universe. This means there is no sampling error, so we use statistical significance as a heuristic guide to the importance of a variable in an equation (computing it as if we had a random sample of MSAs), and interpret results as “pseudo-p-values” to guide our interpretation (as in previous articles: [12], [32], [33]).

Model selection was informed by Akaike information criteria (AIC) and occurred over a number of steps [34]. AIC combines estimation and model selection and is particularly useful when comparing multiple models [35], [36]. Assessing AIC involves comparing AIC values to a minimum AIC or “best” model using the formula: Δ_i_ = AIC_i_−AIC_min_; models having Δ_i_≤2 demonstrate substantial support while models with 4≤Δ_i_≤7 have less support. Models where Δ_i_>10 have essentially no support [36]. AIC was utilized because: 1) it let us compare multiple models in an exploratory way; 2) it let us compare models that were not nested; 3) it helped us identify and avoid overfitted models; while 4) avoiding choice of models that overly-restrict the number of variables included because they use model fit statistics like the Bayes Information Criterion [37] that more heavily penalize extra variables.

Initial analyses used a “quasi-bivariate” approach in which the log of later mortality was modeled as a function of the log of mortality in the earlier period and of each independent variable *seriatim*. The models are bivariate in the sense that they predict the dependent variable from a single substantively-important independent variable, after controlling the initial mortality rate. After organizing independent variables into key domains, separate analyses were run on each domain to determine which set of variables produced the lowest AIC value. We computed AIC for all possible subsets of multiple regression models within each domain for main effects [35]. For each domain, we evaluated all models with Δ_i_≤4 to assess independent variable patterns. Within this range, the best model for each domain was selected by assessing AIC value, parameter estimates, and parsimony.

All variables that appeared in the best domain-specific equations (Table 3) were included in an all-domain exploratory analysis in which AIC was computed for all possible subsets of these independent variables. Model 1 in Table 4 was selected after excluding predictors with additional missing data, such as treatment coverage and counseling and testing coverage. Excluding these variables substantially improved AIC and created a more parsimonious model. Additional models were then constructed by adding these and other variables of theoretical interest with missing data back into the model. Sensitivity analyses were conducted by challenging these models with variables in different forms (for example, by substituting a variable predictor in the form of the difference between early and late periods rather than in the ratio form to see if this improved the AIC). From this process, the best functional form of each variable in the final model was selected. Table 4 presents selected final versions of these models.

**Table 3:** Regression results for domain analyses of variables predicting decline in log mortality rates of IDUs living with AIDS per 10,000 adult population (ages 15–64) - Based on best AIC score within domain.

	N	b	beta	pseudo *p*-value
**Control variables**				
IDUs per 10,000 adult population 1993–95	86	0.002	0.359	0.015
IDUs per 10,000 adult population, Ratio 2000–02 to 1993–95	86	0.718	0.380	<0.001
**Economic Conditions**				
Household Gini 1990	86	4.265	0.233	0.029
**Fiscal Conditions**				
Health Care Expenditure per capita, 1992 (in constant 2010 dollars)	86	−0.001	−0.201	0.058
**Housing**				
Crowding (>1 occupant per room), Difference 2000 to 1990	86	−10.302	−0.283	0.009
**Racial/Ethnic Disparities**				
Black/White Poverty Disparity, Ratio 2000 to 1990	86	0.234	0.157	0.175
Black/White Unemployment Disparity 1990	86	0.085	0.141	0.227
**Interventions**				
Hard Drug Arrests per 10,000 adult population, Ratio 2000–02 to 1993–95	86	0.068	0.268	0.015
HIV Counseling and Testing Coverage for IDU, Ratio 2000–02 to 1993–95	74	−0.191	−0.240	0.035
Drug Treatment Coverage for IDU 1993–95	74	−0.015	−0.137	0.199
**Epidemiologic Factors**				
HIV Prevalence 1993–95	86	0.020	0.381	0.090

**Table 4:** Final regression models exploring the predictors of decline in log mortality rates of IDUs living with AIDS per 10,000 adult population (age 15–64).

	Model 1 (n = 86)		Model 2* (n = 79)		Model 3* (n = 81)		Model 4* (n = 77)	
Independent Variables	b (pseudo *p*-value)	beta	b (pseudo *p*-value)	beta	b (pseudo *p*-value)	beta	b (pseudo *p*-value)	beta
Intercept	−3.243 (<0.001)	0	−3.637 (<0.001)	0	−2.991 (0.003)	0	−3.646 (<0.001)	0
**Control Variables**								
AIDS Mortality (Log), 1993–95	−0.163 (0.001)	−0.358	−0.126 (0.017)	−0.278	−0.178 (<0.001)	−0.385	−0.177 (<0.001)	−0.400
IDUs per 10,000 adult population 1993–95	0.001 (0.041)	0.222	0.003 (0.079)	0.198	0.002 (0.008)	0.308	0.001 (0.031)	0.235
IDUs per 10,000 adult population, Difference in rates 1993–95 to 2000–02	0.005 (0.001)	0.343	0.005 (0.001)	0.341	0.005 (<0.001)	0.407	0.005 (<0.001)	0.424
**Analytic Variables**								
Household Gini 1990	4.587 (0.011)	0.251	5.539 (0.004)	0.297	4.131 (0.028)	0.220	5.722 (0.002)	0.316
Health Care Expenditure per capita 1992 (in constant 2010 dollars)	−0.0013 (0.038)	−0.198	−0.0010 (0.123)	−0.151	−0.0011 (0.087)	−0.161	−0.0013 (0.036)	−0.204
Hard Drug Arrests per 10,000 adult population, Ratio 2000–02 to 1993–95	0.063 (0.010)	0.247	0.061 (0.014)	0.242	0.062 (0.010)	0.245	0.063 (0.006)	0.263
HIV Counseling and Test Coverage for IDU, Difference 2000–02 to 1993–95	–	–	−0.029 (0.037)	−0.217	–	–	–	–
Drug Treatment Coverage for IDU 1993–95	–	–	–	–	−0.022 (0.043)	−0.204	–	–
Methadone Maintenance Coverage for IDU 1993–95	–	–	–	–	–	–	−0.024 (0.162)	−0.140
**AIC** Δ_i_	–	–	−2.874		−2.588		−0.2011	

* Models limited to MSAs where data are not missing on added variable. Change in AIC takes this into account. Given the public health importance of methadone treatment, we estimated Model 4 once it became clear that the more inclusive category, drug treatment coverage, was associated with mortality reduction.

All analyses were conducted using SAS 9.2 software [38].

## Results

Metropolitan areas saw substantial declines in AIDS mortality rates per 10,000 from a median of 0.60 to a median of 0.18, with considerable variation in the extent of decline (Table 1). Descriptive statistics for independent variables appear in Table 2. (Quasi-bivariate regression analyses not shown).

Domain analyses (Table 3) led us to include income inequality, health expenditures, crowded housing, several racial/ethnic disparities variables, hard drug arrests, HIV counseling and testing coverage for IDUs, drug treatment coverage, IDU prevalence rate, and HIV prevalence rate among IDUs in exploratory analyses to develop the final equation.

Table 4, Model 1, presents our final model for MSAs with no missing data (n = 86). It had the lowest AIC value for 86 MSAs. Almost all variables had betas of absolute magnitude 0.20 or above, including the Gini income inequality coefficient for 1990; the ratio of hard drug arrests in 1993–1995 to those in 2000–2002; and the control variables, IDU population prevalence in 1993–1995 and the difference between IDU population prevalence in 2000–2002 minus that in 1993–1995. These variables were associated with higher mortality rates in 2004–2006 and, therefore, to the rate of change in mortality from 1993–1995 to 2004–2006 (with mortality in the earlier period controlled). Health expenditure per capita in 1992 was marginally (−0.198) associated with a greater rate of decline in AIDS mortality over this period.

Additional models, from adding variables singly that reduced the N of MSAs due to missing data, are also presented in Table 4. The Δ_i_ is based on comparing each model AIC to the AIC of Model 1 when we exclude cases with missing data on the added variable. In Models 2, 3 and 4, betas for the difference in counseling and testing coverage between 1993–1995 and 2000–2002 and for baseline drug abuse treatment coverage were <−0.20, indicating that these prevention efforts were associated with lower mortality rates. Methadone maintenance, however, had a weak association (beta = −0.14) with change in mortality rates. Associations of most Model 1 predictors remained relatively constant across all models presented in Table 4; however, the beta for health expenditure dropped below 0.17 if we included either the difference in counseling and testing coverage or baseline drug treatment coverage.

It should be noted that when Model 1 was run for the subset of metropolitan areas that had data available for counseling and testing, and, separately, drug abuse treatment, the pseudo-p values for health expenditures per capita increased above 0.05.

To test whether these results might differ for MSAs that had the worst initial AIDS epidemics, we conducted stratified analyses for the 28 MSAs with the worst epidemics (defined by having AIDS mortality rates in the 1993–1995 period greater than 0.75 per 10,000 adult population) and separately for the other 58 MSAs. This stratification point was derived by inspection of the distribution of mortality rates in the earlier period. The lowest 58 MSAs all had low rates, and there was a modest-size gap in mortality rates between the highest rate in the lower group and the lowest mortality rate in the higher group. When the models in Table 4 were run for the 58 MSAs with lowest initial mortality among IDUs with AIDS, the results were very similar to those in Table 4. In the subset of 28 MSAs with the highest mortality rates, there was a reversal in direction of the coefficient for the ratio of hard drug arrests in 2000–2002 to those in 1993–1995. In these MSAs, a higher increase in arrest rates was associated with a decrease in mortality rates. To test whether this might be a multicollinearity effect, we examined the quasi-bivariate for the arrest ratio, but here too a higher increase in arrest rates was associated with a decrease in mortality rates.

## Discussion

Mortality rates among IDUs with AIDS decreased greatly in most MSAs following the introduction of HAART. The rate of this decline, however, varied dramatically: mortality rates fell to less than one-fifth of their former values in three MSAs but remained at 70% or more in three others.

Pre-HAART income inequality was associated with higher mortality in the HAART era. Although past research has found income inequality to be associated with higher rates of some causes of mortality [39], [40], [41], mechanisms through which income inequality would slow the decline in mortality among IDUs with AIDS are not clear. The literatures on social determinants of health and risk environments suggest strong associations between income (or other economic) inequality and stress levels [42], [43], [44], and neighborhood-level income inequality has been found to be related to drug users' overdose death rates with mediators that suggest that the pathway between inequality and overdose mortality may include stress [45]. More research should be conducted on whether and how such mechanisms might lead to a greater propensity among the population to begin injecting drugs or to do so in high-risk ways and whether income inequality reduces (or results from reduced) social cohesion leading to less investment in public healthcare or other services that IDUs use to prolong life [46]. Causation might be specific to IDUs or people living with AIDS, which suggests that parallel analyses might be conducted for IDUs without AIDS or non-IDUs with AIDS. Since greater public expenditures for health in an MSA were associated with greater declines in mortality among IDUs living with AIDS, future cutbacks in general public health expenditures might increase mortality—and perhaps also that of non-IDUs living with AIDS.

Drug abuse treatment and HIV counseling and testing rates had positive impacts on mortality among IDUs living with AIDS. In sensitivity analyses using different forms of these variables, however, these findings were not very stable, which reduces our confidence in these results—as does the lack of association of methadone treatment variables with mortality. These unstable results may be the result of relatively low coverage rates of these services [33], [47]. Neither overdose death rates nor changes in these rates predicted changes in mortality rates among IDUs living with AIDS.

We have previously shown that heroin/cocaine possession arrests do not reduce the number of IDUs per capita, and are associated with higher subsequent HIV seroprevalence among IDUs [8], [13]. In this analysis, we found that increases in hard drug arrests were associated with slower decreases in death rates in most MSAs.

We also found that increases in hard drug arrests were associated with more rapid decreases in death rates for the 28 MSAs that had the highest mortality rates among IDUs living with AIDS in the 1993–1995 period. Our data do not allow us to come to any definitive conclusions about what is happening, but we note that the five MSAs that had the highest early mortality rates—New York, Jersey City, Newark, Baltimore and San Francisco—had considerably higher early mortality than the next highest MSA and also were five of the six highest MSAs (among the 28) in hard drug arrest rates in 2000–2002. These data are consistent with either of two somewhat-related explanations: 1) It may be that these MSAs, given the severity of their AIDS epidemics and AIDS mortality rates early in the epidemic, took particular care to make sure that arrestees and prisoners with HIV received good medical care while in custody. 2) It also might be the case that these MSAs did a particularly good job of making HIV care available outside of custodial settings. Both of these explanations are consistent with the findings in the regression models given the fact that arrest rates remained high in these MSAs.

Overall, then, we do not know the mechanisms through which hard drug arrests might influence mortality among IDUs with AIDS. Such mechanisms might include disruptions in ART treatment for IDUs who get arrested in some MSAs, whether at time of arrest, while incarcerated, or upon release [48]; stress from arrest or imprisonment; or exposure to pathogens while incarcerated [49], [50]. Alternatively, a higher risk of hard drug arrest might increase stress among IDUs in the community and/or lead them to hide their drug use by injecting in settings where they might be exposed to other pathogens or to risk STI or homicide by engaging in sex trading [51], [52], [53], [54], [55], [56], [57]. Arrests might also lead to greater HIV transmission, and subsequent mortality, by causing higher rates of risk behavior, greater turnover in injection and sexual networks, or increased partner concurrency, all of which could increase the transmission of HIV at a community level [58], [59]. As noted above, these relationships might work differently in the MSAs most affected by AIDS in the earlier period.

These findings are subject to several limitations. Causal mechanisms are hard to study at a single level of analysis since both higher-level and lower-level variables may affect observed relationships. Care must be taken both to avoid interpretations that fall into the ecological fallacy and also to be open to interpretations that are valid at a single level of analysis—in this case, at the MSA level. Here, we are limited in our ability to study pathways by which independent variables lead to outcomes. As with many studies, including many on social determinants of health [60], [61], [62], we cannot specify the mechanisms by which differences in MSA characteristics, like hard drug arrest rates or crowded housing, or how effects of interventions on HIV incidence and the effect of ART on reducing the rate of AIDS incidence among the HIV-infected, are associated with a slower decline in mortality among IDUs with AIDS at the individual level. In addition, all variables are subject to measurement error, which may be considerable for arrest data, drug abuse treatment and HIV counseling and testing coverage data. We are seriously limited by unavailability of data on ART use and adherence rates among IDUs in these MSAs.

This paper is exploratory in spite of being guided by ecosocial, risk environmental and dialectical theory. These theories provided little guidance about which functional form of change measures (differences or ratios) to use in multivariable models. These theories also suggested far more independent variables of importance than could be included in one equation. Thus, these results might have been shaped by our exploratory procedures to determine which variables to retain in the model. (Our sensitivity analyses which challenged Equation 2 with additional variables provide some protection against errors here.) Our analyses thus generated rather than tested hypotheses. Since this is a study of the set of the largest metropolitan areas in the US, rather than of a partial sampling of MSAs, it is not possible to draw a new sample to test these hypotheses. Instead, researchers might investigate whether future changes in these variables are followed by changes in mortality among IDUs living with AIDS in the predicted directions and magnitudes. Testing their generalizability to other countries and to smaller MSAs or counties in the US would also be useful.

Guided both by the findings of this paper and also by our prior findings that income inequality helped to predict the MSA-level population prevalence of IDUs, HIV prevalence among IDUs, and HIV incidence among IDUs in 1992 [11], we hypothesize that income inequality may be a particularly important ecosocial variable in shaping HIV epidemics among people who inject drugs. We also hypothesize that the extent of coverage by programs like HIV counseling and testing and drug abuse treatment, as well as the importance of arrest rates for hard drug use, point to the dialectical importance of political and budgetary decisions for the epidemic [3], [4], [5], [6].

Mortality among people who inject drugs might be decreased by reducing metropolitan income inequality and increasing public expenditures on health (including HIV-related care) [63]. Increasing availability of public health interventions like drug abuse treatment and HIV counseling and testing might reduce mortality among injectors living with AIDS. Given prior evidence that drug-related arrest rates are associated with higher HIV prevalence rates among IDUs and do not seem to decrease IDU population prevalence [8], changes in laws and policing practices to reduce such arrests while still protecting public order should be considered alongside increasing public health intervention coverage.
